# Comparative Analysis of Chloroplast Genomes Reveals Molecular Evolution and Phylogenetic Relationships in *Fraxinus* (*Fraxinus mandshurica*)

**DOI:** 10.3390/ijms27177939

**Published:** 2026-09-06

**Authors:** Wenxuan Liu, Jiawei Wu, Haonan Zheng, Guanzheng Qu, Sui Wang

**Affiliations:** 1 State Key Laboratory of Tree Genetics and Breeding, Northeast Forestry University, 26 Hexing Road, Harbin 150040, China; liuwenxuan@nefu.edu.cn (W.L.); wjwwjw152101@126.com (J.W.); zhnzhn0123@163.com (H.Z.); 2 Key Laboratory of Saline-Alkali Vegetation Ecology Restoration, Ministry of Education, Northeast Forestry University, Harbin 150040, China; 3 National Key Laboratory of Smart Farm Technologies and Systems, Northeast Agricultural University, No. 600 Changjiang Road, Harbin 150030, China

**Keywords:** *Fraxinus mandshurica*, chloroplast genome, phylogenetic analysis, selection pressure, nucleotide diversity

## Abstract

*Fraxinus mandshurica* (Manchurian ash) is an ecologically and economically valuable hardwood tree native to Northeast Asia, yet its genomic resources remain limited. We assembled its complete chloroplast (cp) genome (155,559 bp) using hybrid PacBio and Illumina sequencing and performed comparative, phylogenetic, and evolutionary analyses. The cp genome exhibits a typical quadripartite structure encoding 132 gene copies, comprising 114 unique genes (80 protein-coding, 30 tRNA, and 4 rRNA genes), with 18 genes duplicated in the inverted repeat (IR) regions. Simple sequence repeat analysis revealed dominance of mononucleotide A/T repeats. Phylogenetic analysis of 53 complete cp genomes strongly supported the monophyly of *Oleaceae* and resolved *F. mandshurica* as sister to the North American *F. nigra*, consistent with previously proposed Miocene intercontinental dispersal scenarios between East Asia and North America. Most protein-coding genes were under strong purifying selection (Ka/Ks << 1), whereas *petB*, *rpl2*, and several *ndh* genes showed elevated Ka/Ks values that are suggestive of altered selective constraint but are based on very few substitutions and are therefore not, on their own, evidence of positive selection. Nucleotide diversity (Pi) analysis identified 15 hypervariable intergenic spacers (mean Pi = 0.067), among which *trnM-CAU-rps14*, *ndhJ-ndhK*, and *petL-petG* represent promising candidate barcode regions requiring further validation. This study provides a high-quality, fully annotated cp genome of *F. mandshurica* and a valuable genomic resource for future phylogenetic, population genetic, and conservation studies of this important genus.

## 1. Introduction

*Fraxinus* (ash) is a genus of approximately 43 species of deciduous or evergreen trees and shrubs distributed across the Northern Hemisphere [1]. Ash species are of outstanding ecological and economic importance, yet many have been severely threatened in recent decades by invasive pests and pathogens, most notably the emerald ash borer (*Agrilus planipennis*) in North America [2] and ash dieback (*Hymenoscyphus fraxineus*) in Europe [3]. Among them, *Fraxinus mandshurica* Rupr. (Manchurian ash) is one of the most cold-tolerant species of the genus and reaches the northernmost limit of ash distribution in northeastern Asia. In Northeast China, it is one of the “Three Hardwood” species (*F. mandshurica*, *Juglans mandshurica*, and *Phellodendron amurense*) and is economically significant for its hard, beautifully textured timber used in furniture, flooring and building, as well as for its bark (“qinpi”, Cortex fraxini), which is used as a traditional Chinese medicine containing compounds with immunosuppressive activity [4]. Because of long-term overexploitation and habitat loss, natural populations of *F. mandshurica* have declined sharply, and the species is now listed as a national second-class protected wild plant in China [5], making the characterization of its genetic resources an urgent priority for conservation and sustainable utilization.

In recent years, genomic resources in *Fraxinus* have grown rapidly. A chromosome-scale genome is available for European ash (*F. excelsior*) [6], and chromosome-scale genome assemblies have been reported for several other species [7]. At the plastome level, complete chloroplast genomes have been assembled for multiple *Fraxinus* species, and comparative analyses have improved species identification and phylogenetic resolution within the genus [8]. However, plastid genomic information for *F. mandshurica* remains extremely limited: no fully annotated chloroplast genome has been reported for this species, and most existing studies focus on physiology, biochemistry, silviculture and tissue culture [9]. This gap constrains several research directions of direct relevance: (i) the molecular authentication of “qinpi” against adulterants using plastid barcodes; (ii) phylogeographic and population genetic studies of a species occupying the cold margin of the genus range, where chloroplast markers with predominantly maternal inheritance can trace glacial refugia and post-glacial expansion; and (iii) conservation genomics of a threatened timber species. Moreover, as a native Asian host that has co-evolved with *H. fraxineus*, *F. mandshurica* shows greater tolerance to ash dieback than European species [10], so its genetic resources are also of interest for breeding ash dieback resistance.

Chloroplast genomes are typically 120–160 kb in length with a conserved quadripartite structure, in which two inverted repeats (IRs) separate the large and small single-copy regions (LSC and SSC), and contain approximately 100–130 genes [11,12]. Owing to their predominantly maternal inheritance [13], moderate substitution rates, and the presence of both conserved and variable regions, chloroplast genomes are powerful and cost-effective markers for species identification [14], phylogenetic reconstruction and population genetics [8,11]. For a species such as *F. mandshurica* that still lacks extensive nuclear genomic infrastructure, the chloroplast genome offers an immediately accessible first step toward understanding its evolutionary history and genetic resources.

In this study, we assembled and annotated the complete chloroplast genome of *F. mandshurica* from whole-genome sequencing data, and characterized its genome structure, gene content, repetitive sequences and codon usage. We further reconstructed the phylogenetic position of *F. mandshurica* within *Oleaceae* based on complete plastome sequences. These results provide a fundamental genomic resource for species authentication, population genetics and conservation of *F. mandshurica*, and contribute to a more complete picture of plastome evolution in *Fraxinus*.

## 2. Results

### 2.1. Chloroplast Genome Assembly

The complete chloroplast genome of *F. mandshurica* was assembled as a single circular molecule of 155,559 bp, with an overall GC content of 37.82%. The genome exhibits the typical quadripartite structure of angiosperm cp genomes, comprising a large single-copy (LSC) region of 86,429 bp, a small single-copy (SSC) region of 17,850 bp, and two inverted repeat (IR) copies of 25,640 bp each (IRa and IRb). The two IR copies were 100% identical inverted repeats, and the IR boundaries were localized at positions 86,418/112,058 (IRa) and 129,908/155,548 (IRb). Validation of the assembly with both short- and long-read data confirmed its high quality. Re-mapping of the Illumina reads (bwa mem) placed 9,169,356 of 579,112,560 reads (1.58%) onto the chloroplast genome, corresponding to a mean coverage of 7043× and complete (100%) breadth of coverage across the entire genome. Sequencing depth was uniform across the LSC, SSC, and IR regions (mean depth 6885.20×, 5594.30× and 7813.70×, respectively), with the IR copies showing elevated depth relative to the single-copy regions (approximately 1.1–1.4×), as expected for duplicated regions. All four IR/SC junctions were independently supported by PacBio long reads (340–6466 uniquely mapped reads per junction), and 1890 PacBio reads spanned the linearization point, providing direct evidence for the circularity of the assembled molecule.

**Figure 1:**
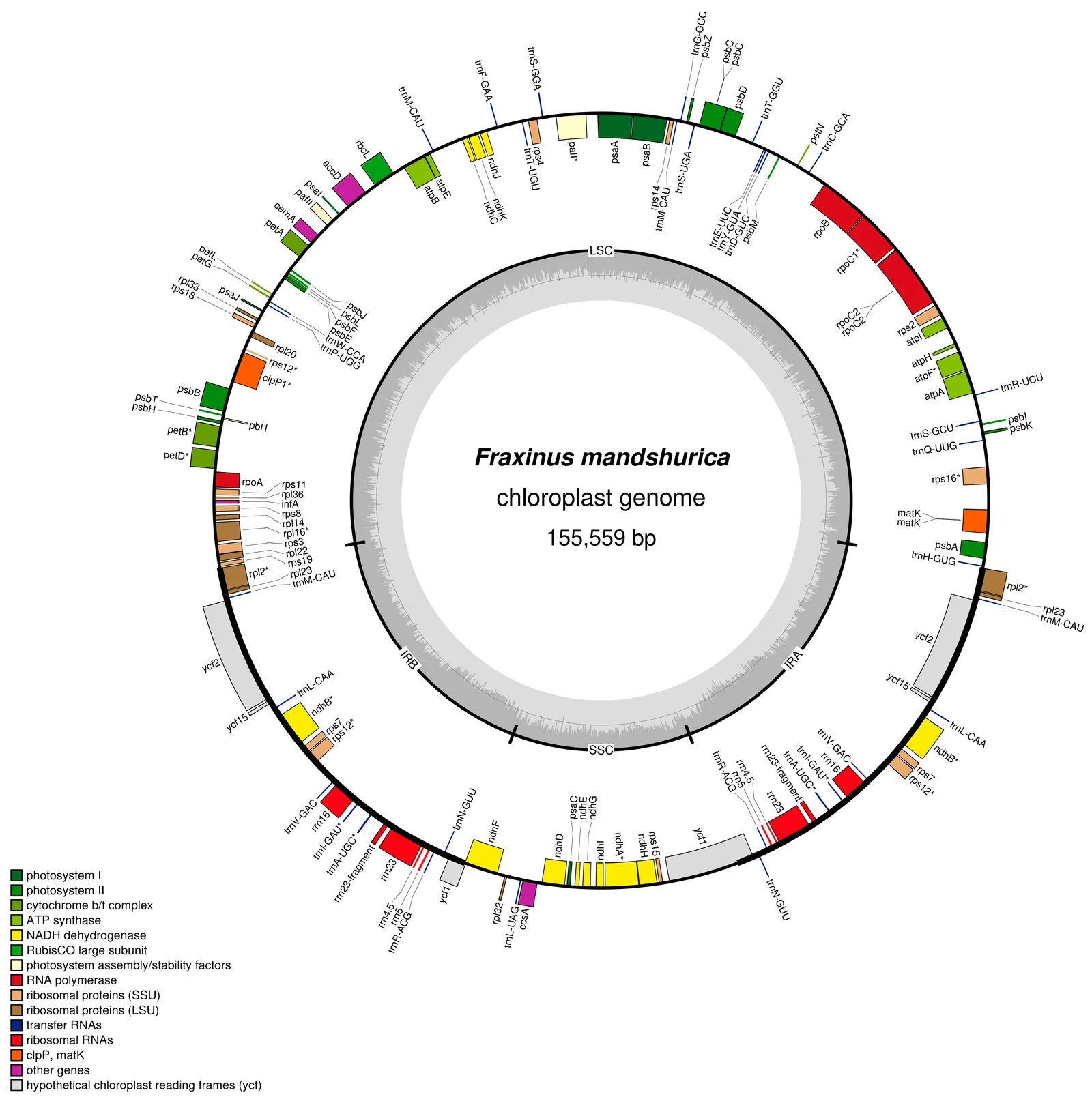
Circular map of chloroplast genome of *F. mandshurica* with annotated genes. The different functional genes groups are shown in different colors, which are shown on the bottom left. The genes transcribed in clockwise and counterclockwise are shown inside and outside of the external circle, respectively. The inner circle represents that the quadripartite structure contains two copies of the inverted repeat (IR) region (IRA and IRB), which separate large single-copy (LSC) and small single-copy (SSC) regions. The dark gray color of inner circle shows the GC content, and AT content in light gray.

The complete chloroplast genome sequence of *F. mandshurica* has a total length of 155,559 base pairs (bp). Like most land plants, the circular cpDNA has a typical quadripartite structure. A large single-copy (LSC) region of 86,429 bp separated from a 17,850 bp small single-copy (SSC) region by two inverted repeats (IRs), each of 25,640 bp. The GC content of the whole genome is 37.82%, and the GC contents of the LSC, SSC and IR regions are 35.81%, 31.96% and 43.23%, respectively. Obviously, the IRs are relatively GC-rich regions (Figure 1).

### 2.2. Chloroplast Genome Annotation

We annotate a total of 132 gene copies in the *F. mandshurica* cp genome, of which 18 are duplicated in the IR regions and therefore correspond to 114 unique genes. Among these 114 unique genes, there are 80 protein-coding genes, 30 tRNA genes, and 4 rRNA genes. The GC content of different types of genes varied greatly; it is higher in the rRNAs (55.28%) and tRNAs (52.80%) than in the protein-coding genes (37.82%). Although most of these genes are single-exon genes, 15 genes (6 tRNA, 9 mRNA) contain 2 exons and 3 mRNA genes contain 3 exons (Figure 1).

Among the *F. mandshurica* cp genes, several are unusual with special structure. The *rps12* gene which codes for the homologous ribosomal protein S12 is a trans-spliced gene [15], three exons of which are located in the LSC region and IR regions, respectively. Unlike ordinary genes using ATG as their initiation codons, there are 3 *F. mandshurica* cp genes that have unusual initiation codons, such as ACG in *ndhD* and GTG in *rps19*, *ycf15*; these genes code for a variety of proteins involved in photosynthesis, transcription and translation. Table 1 shows the gene functions and groups in the *F. mandshurica* cp genome.

### 2.3. Codon Usage Bias and RSCU Value Statistics

Codon usage bias refers to differences in the frequency of occurrence of synonymous codons in coding DNA. Although codon degeneracy manifests as the multiplicity of three-base-pair codon combinations that specify an amino acid, synonymous codon usage frequency is always different, which can be measured by RSCU value. To some extent, this is influenced by mutational bias coupled with both natural selection for translational optimization and genetic drift [16]. There were 80 unique protein-coding genes in the *F. mandshurica* cp genome, including 26,485 codons in total. Genes duplicated in the IR regions were retained in the codon-usage analysis because they are actively transcribed and therefore contribute to the total codon pool, consistent with previous codon-usage surveys of Oleaceae plastomes. The three amino acids with the highest proportions were leucine (10.52%), isoleucine (8.48%) and serine (7.83%), while the lowest proportions were observed for cysteine (1.10%), tryptophan (1.77%) and the stop codons (0.33%). Actually, this codon usage bias is highly conserved among Oleaceae species [17]. Table 2 showed the RSCU value in *F. mandshurica* and some other species cp genes. Compared with the distantly related *Nicotiana tabacum*, the value of *F. mandshurica* is closer to that of *Forsythia suspensa*, but the differences were not obvious, especially the trend was completely consistent (greater than 1.00, less than 1.00 or equal to 1.00).

Here we used RNA sequences without editing to compute the codon usage and RSCU values; because RNA editing was not incorporated, the values at edited sites may differ slightly from the protein-level codon usage.

### 2.4. Comparison of Chloroplast Genome Sequences with Those of Other Species

**Figure 2:**
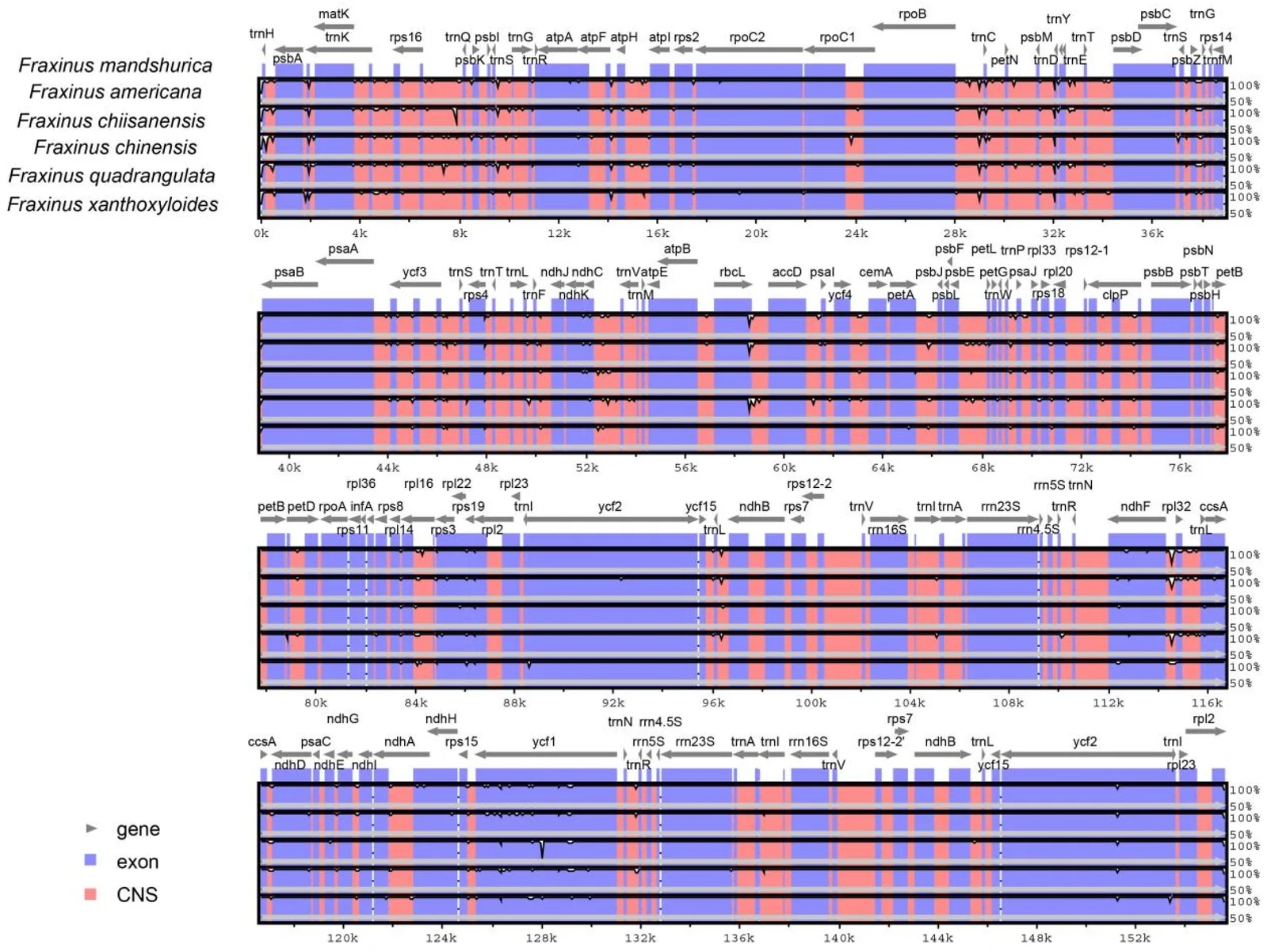
Sequence alignment of 6 cp genomes in the genus *Fraxinus* using the mVISTA program with the gene annotation of *F. mandshurica* as a reference. Grey arrows above the alignment indicate the transcriptional directions of genes. Genome regions are color-coded as exon and conserved non-coding sequences (CNS). The RankVISTA probability threshold is set at 0.5. The *Y*-axis indicates the percent identity between 50 and 100%.

Despite high conservation of cp genome sequences, especially among closely related species, there are also some hotspots of variation on these sequences which can be used for species classification, phylogenetic analysis and domestication studies. In order to visualize the similarities and differences in the cp genome sequences among *Fraxinus* spp., we selected the *F. mandshurica* cp genome sequence we assembled and 5 other *Fraxinus* spp. cp genome sequences downloaded from NCBI organelle genome database for sequence alignment. These species are each from 6 of the 7 sections of the genus *Fraxinus* (the other section has no complete cp genome sequences in the NCBI database), which can represent the variation regions among different sections. The mVISTA plot was used to compare these *Fraxinus* spp. cp genome sequences with the gene annotation of *F. mandshurica* as a reference (Figure 2). Overall, these *Fraxinus* spp. cp genomes had little difference in size, ranging from 155,541 bp to 155,694 bp. Figure 2 shows that the structures of all the six *Fraxinus* spp. cp genomes are conserved and no obvious translocations or inversions are detected. Similar to previous studies, these sequences have a high level of overall similarity, especially in the coding region, but some hotspots of variation also exist on the sequences. Generally speaking, the most highly polymorphic regions were located in the intergenic regions (such as *rpl2*—*trnH*-*GUG*, *rps16*—*trnQ*-*UUG*, *trnS*-*GCU*—*trnG*-*UCC*, *trnE*-*UUC*—*trnT*-*GGU*, *psbE*—*petL*, *rpl32*—*trnL*-*UAG*). However, the ycf1 gene which had higher variability regions is an exception.

### 2.5. SSR Identification and Analysis

**Figure 3:**
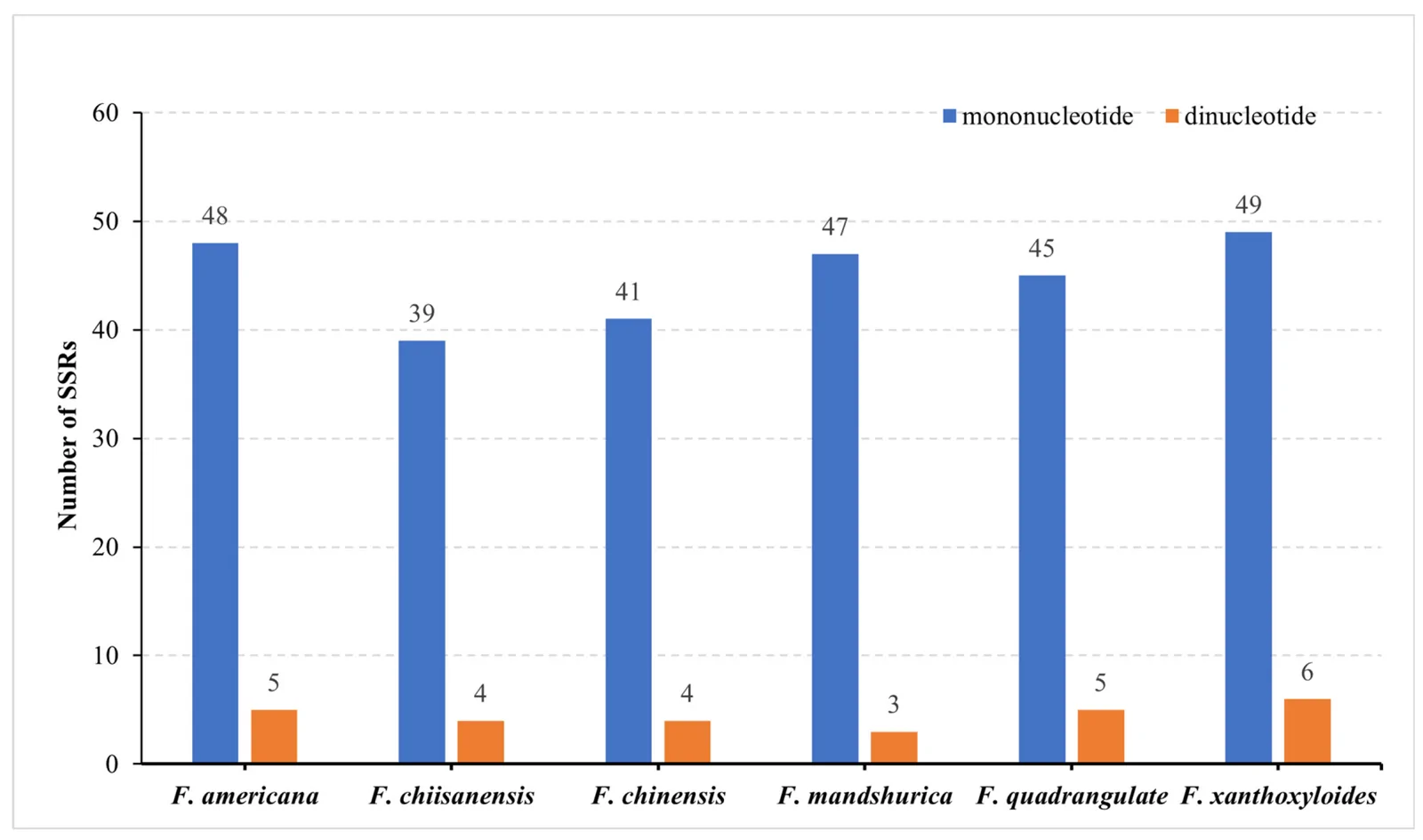
Number and type of simple sequence repeats (SSRs) identified in the chloroplast genomes of six *Fraxinus* species.

SSRs identification is another standard analysis for cp genome analysis. Due to the existence of interspecific variation, this sequence is very useful in species classification, genome evolution and rearrangement [18,19,20]. In this study, we only focused on species representative from different sections of the *Fraxinus* genus. A total of 53, 43, 45, 50, 50 and 55 SSRs were detected in the *F. americana*, *F. chiisanensis*, *F. chinensis*, *F. mandshurica*, *F. quadrangulata* and *F. xanthoxyloides* cp genomes, respectively (Figure 3). Among the SSRs, most of them were mononucleotide repeats and few were dinucleotide repeats. No tri-, tetra-, penta- and hexanucleotides repeats were found in all the six *Fraxinus* spp. cp genomes. For *F. mandshurica*, considering sequence complementary, the mononucleotides were composed of 46 A/T and 1 C/G, and the dinucleotides were composed of 1 AG/CT and 2 AT/AT. To develop lineage-specific markers, CandiSSR was used to identify candidate polymorphic SSRs. Unfortunately, no candidate SSRs were identified among the *Fraxinus* species. Because only one plastome per species was examined, this result primarily reflects interspecific variation and does not directly evaluate population-level marker utility.

### 2.6. IR Expansion and Contraction

**Figure 4:**
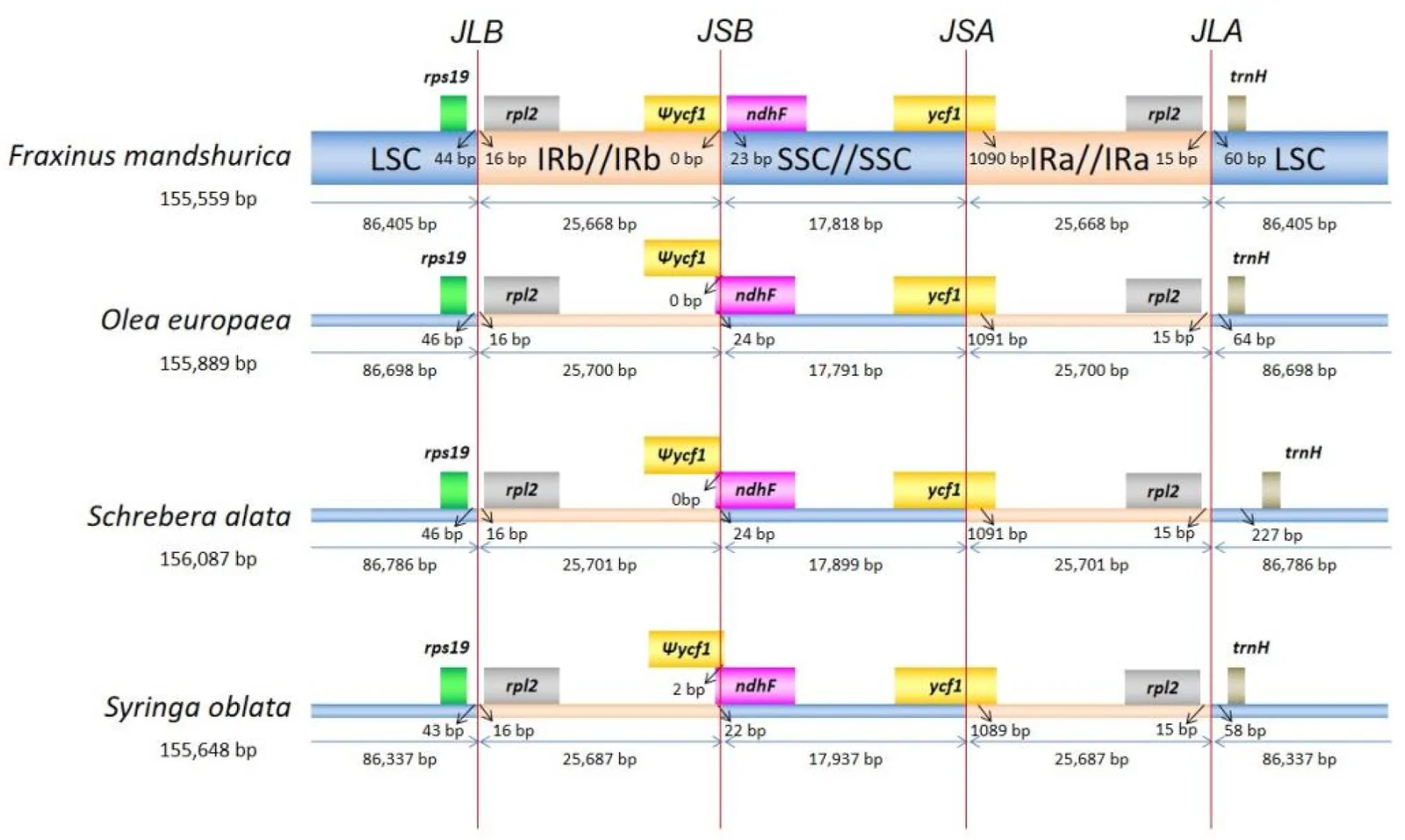
Representative comparison of IR/SC boundary regions of Oleaceae chloroplast genomes. The thin vertical lines represent the junction of each region, and the map displays information about the genes near the junction. LSC, Large single copy; SSC, Small single copy; IRa and IRb, inverted repeats. JLB, junction between LSC and IRb; JSB, junction between SSC and IRb; JSA, junction between SSC and IRa; JLA, junction between LSC and IRa.

Size variation in cp genomes is partly a result of expansion and contraction at the borders of the IR regions. The differences in IRs also reflect phylogenetic history. Here, we selected four representative species in *Oleaceae* and a detailed comparison of four junctions, LSC/IRA (JLA), LSC/IRB (JLB), SSC/IRA (JSA) and SSC/IRB (JSB), between the two IRs (IRA and IRB) and the two single–copy regions (LSC and SSC), was completed among *Fraxinus mandshurica*, *Olea europaea*, *Schrebera alata*, and *Syringa oblata* (Figure 4). The SSC/IRA junction is located in the *ycf1* region in all *Oleaceae* species chloroplast genomes and extends a different length into the SSC region in all genomes; the IRB region includes 1090 bp (*F. mandshurica*), 1091 bp (*O. europaea*), 1091 bp (*S. alata*), and 1089 bp (*S. oblata*) of the ycf1 gene, respectively. Similarly, the *trnH* gene is located in the LSC region, 60, 64, 227, and 58 bp away from the IRA/LSC border in the four *Oleaceae* chloroplast genomes, respectively. The *ψycf1* pseudogene was present in all four genomes: in *S. oblata* it extended several base pairs into the SSC region, whereas in the other three species it was entirely located within the IRb region. The distance between the *ψycf1* pseudogene and the *ndhF* gene was similar in the four genomes (about 23 bp). The *rps19* gene is located in the LSC region 44, 46, 46, and 43 bp away from the LSC-IRa border in these four *Oleaceae* chloroplast genomes, respectively. The *rpl2* gene is located in the IR regions, and the IRa and IRb regions each contain 16 bp of the *rpl2* gene.

### 2.7. Phylogenetic Relationships

**Figure 5:**
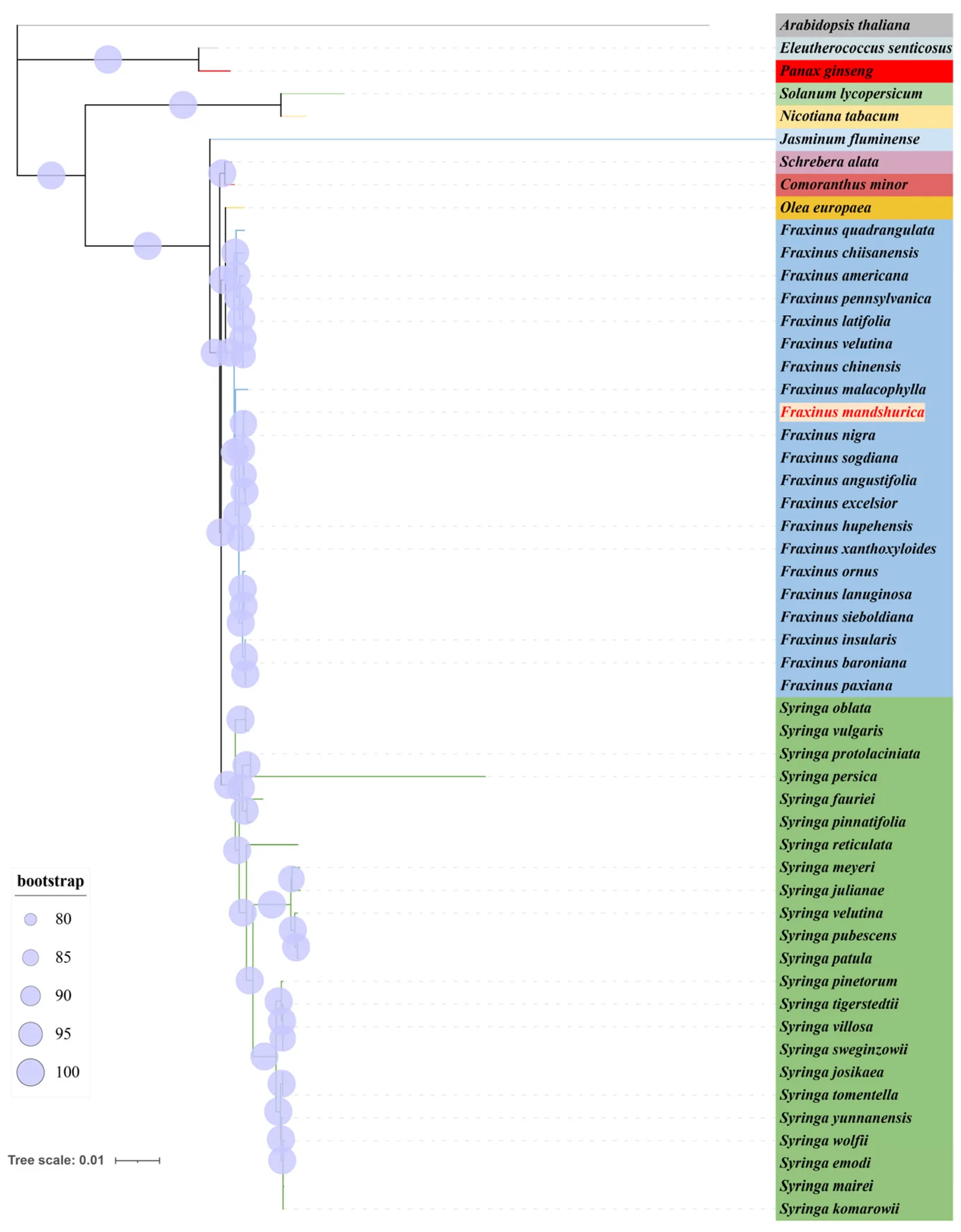
Phylogenetic tree constructed based on chloroplast genome sequences.

The maximum likelihood phylogenetic tree reconstructed from complete chloroplast genome sequences clearly demonstrates the evolutionary topology of the family Oleaceae and its related taxa (Figure 5). To rigorously root the tree, distantly related species including *Solanum lycopersicum*, *Nicotiana tabacum*, *Arabidopsis thaliana*, as well as Apiales species *Eleutherococcus senticosus* (a medicinal plant) and *Panax ginseng*, were selected as outgroups. This strategic choice not only stabilizes the topological direction at the root but also provides a macroscopic baseline for future comparisons of secondary metabolite evolution in medicinal plants from a broader angiosperm perspective (encompassing orders such as Apiales and Lamiales). The results show that all Oleaceae species cluster into a highly supported monophyletic group.

The phylogenetic topology recovered in this study is broadly congruent with previous molecular phylogenetic frameworks of Oleaceae, although some differences are evident in the relationships among major lineages. Within the taxa sampled here, *Jasminum fluminense* was recovered as the earliest-diverging lineage, followed by a strongly supported clade comprising *Schrebera alata* and *Comoranthus minor*. The close relationship between *Schrebera* and *Comoranthus* is consistent with their placement within Schreberinae in previous molecular classifications of Oleaceae [21]. *Olea europaea* diverged subsequently, whereas the sampled species of *Fraxinus* and *Syringa* each formed well-supported monophyletic groups and were recovered as sister lineages in the present plastome-based analysis. Previous phylogenetic studies incorporating evidence from both nuclear and organellar genomes have generally supported the major tribal and subtribal framework of Oleaceae, while also revealing some discordance among genomic datasets in the resolution of deeper relationships [22]. Therefore, rather than indicating complete agreement with all previously proposed topologies, our results provide additional plastome-scale evidence for the relationships among the sampled Oleaceae taxa and demonstrate the strong phylogenetic signal of complete chloroplast genomes for resolving relationships within this taxonomic sampling.

Within the genus *Fraxinus*, our finding that *Fraxinus mandshurica* and the North American endemic species *Fraxinus nigra* form a tight sister group carries profound biogeographical significance. They further form a highly stable evolutionary branch with other species of the section *Fraxinus*, including *Fraxinus sogdiana*, *Fraxinus angustifolia*, and *Fraxinus excelsior*. This specific grouping precisely reflects the traditional taxonomic classification of the section *Fraxinus*. The close sister relationship between *F. mandshurica* (East Asia) and *F. nigra* (North America) is highly consistent with the classic study by Hinsinger and colleagues [23] on the phylogeny and biogeographic history of *Fraxinus* who noted that both species belong to section *Fraxinus* and confirmed their close relationship at the molecular level. This East Asian–North American sister relationship is consistent with previously proposed Miocene intercontinental dispersal and subsequent vicariance scenarios between Eurasia and North America; however, divergence-time estimation and ancestral-area reconstruction would be required to test this hypothesis directly. The present phylogenetic tree recovers this intercontinental phylogenetic link, underscoring the value of complete chloroplast genomes for resolving both deep and shallow evolutionary relationships within Oleaceae.

### 2.8. Selection Pressure and Evolutionary Dynamics

**Figure 6:**
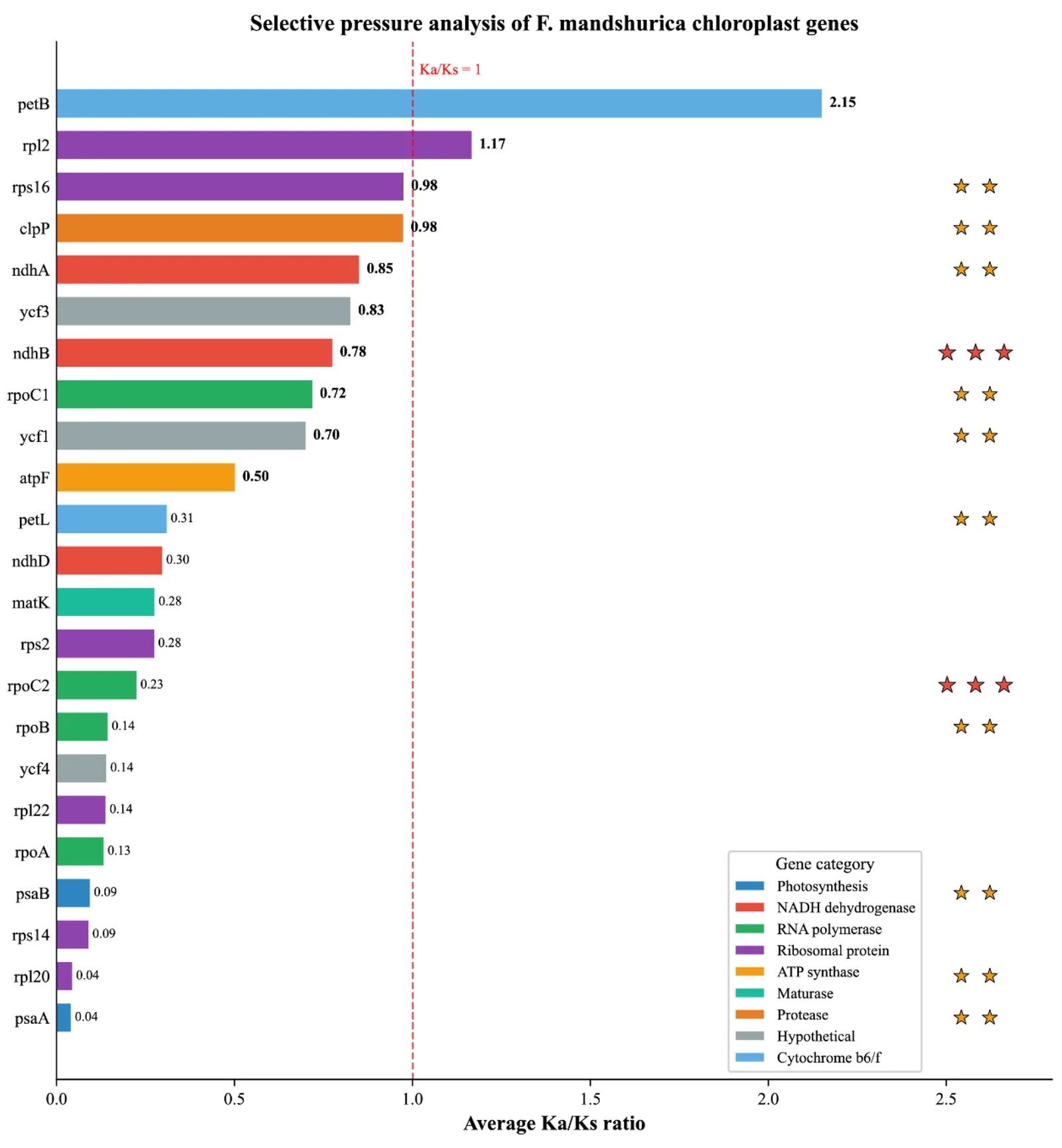
Selective pressure analysis of *F. mandshurica* chloroplast protein-coding genes. Horizontal bar chart showing average Ka/Ks ratios across *F. mandshurica* orthologous genes compared with *F. nigra*, *F. excelsior*, and *F. americana*. Genes are color-coded by functional category. The red dashed line at Ka/Ks = 1.0 marks the neutral evolution threshold. Red stars (three stars) indicate genes with valid Ka/Ks values in all three pairwise comparisons, while orange stars (two stars) denote genes with valid Ka/Ks in two of the three species comparisons.

To assess the selective constraints acting on protein-coding genes of the *F. mandshurica* cp genome, we computed pairwise Ka/Ks ratios against three closely related *Fraxinus* species (*F. nigra*, *F. excelsior*, and *F. americana*). The analysis included 80 unique protein-coding genes, of which 14 to 20 yielded valid Ka/Ks values per species comparison (Figure 6, Table 3), after genes lacking synonymous substitutions (Ks = 0) or without a reliable single-copy orthologous alignment were excluded.

Across all three pairwise comparisons, the vast majority of chloroplast genes exhibited Ka/Ks values considerably below 1, indicating strong purifying selection and high functional constraint, consistent with the overall conservation of cp genomes in Oleaceae. Photosystem-associated genes (e.g., *psaA*, *psbA*, *psbB*, *psbI*), most ribosomal protein genes (e.g., *rps2*, *rpl20*, *rps14*), and components of the cytochrome b6/f complex (e.g., *petN*, *petG*) showed Ka/Ks ratios close to zero, reflecting their essential roles in photosynthesis and translation.

Notably, several genes displayed elevated Ka/Ks ratios suggestive of relaxed or potential diversifying selection (Table 3). (i) *petB* (cytochrome b6/f complex subunit) and *rpl2* (ribosomal large subunit protein L2) showed high Ka/Ks estimates in the *F. excelsior* comparison (2.15 and 1.17, respectively). However, because these estimates were based on only one valid species comparison, they were treated as exploratory observations rather than robust evidence of altered selection. (ii) *rps16* (ribosomal small subunit S16), with Ka/Ks = 0.92–1.04 in both *F. excelsior* and *F. americana*; (iii) *clpP* (ATP-dependent Clp protease proteolytic subunit), with Ka/Ks = 1.04 in *F. excelsior* and 0.91 in *F. americana*; (iv) *ndhA* and *ndhB* (NADH dehydrogenase subunits), which exhibited consistently elevated Ka/Ks across species pairs (0.66–0.85 for *ndhB* in all three comparisons; 0.82–0.89 for *ndhA* in two comparisons); and (v) *ycf1* (hypothetical chloroplast ORF 1), with Ka/Ks = 0.62–0.78. The recurrent detection of elevated Ka/Ks in the *ndh* genes (*ndhB* in all three pairwise comparisons and *ndhA* in two) is particularly noteworthy, as the NDH complex plays a crucial role in chloroplast cyclic electron flow and redox regulation. Although *ndhB* and *ycf1* are below the 0.8 highlight threshold, they are listed in Table 3 for completeness.

### 2.9. Nucleotide Diversity and Hypervariable Regions

**Figure 7:**
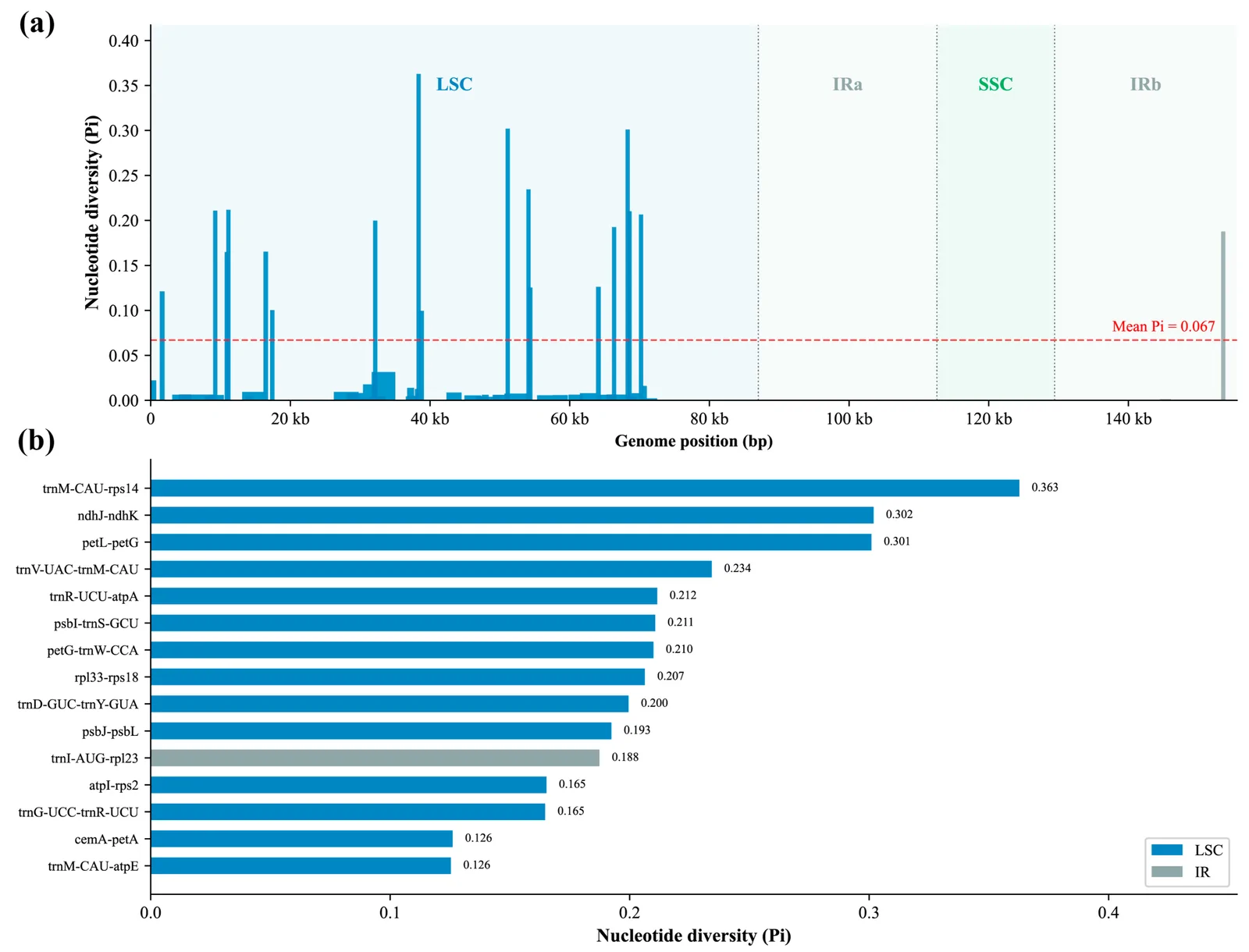
Nucleotide diversity (Pi) analysis of *Fraxinus* chloroplast genomes. (**a**) Genome-wide distribution of nucleotide diversity across 15 species of *Fraxinus*. The LSC, IRa, SSC, and IRb regions are indicated. The red dashed line shows the mean Pi (0.067). (**b**) The 15 most variable intergenic spacer regions ranked by Pi. Bars are color-coded by genomic region (LSC, blue; IR, gray). Flanking gene names are indicated on the *y*-axis.

To gain further insight into the patterns of sequence variation across the genus *Fraxinus* and to identify candidate molecular markers for downstream taxonomic and population genetic studies, we quantified nucleotide diversity (Pi) across 57 intergenic spacer regions of the *F. mandshurica* cp genome using orthologous sequences from 15 *Fraxinus* species. The genome-wide mean Pi across all analyzed intergenic spacers was 0.067, ranging from 0 to 0.363, indicating substantial heterogeneity in evolutionary rates across the cp genome (Figure 7, Table 4). The number of species contributing to individual intergenic spacers ranged from 4 to 14 (Table 4), and this variation in representation was taken into account when hypervariable regions were ranked.

Consistent with the conserved nature of plastome inverted repeat (IR) regions, Pi within both IR copies was extremely low (essentially zero), reflecting strong sequence homogenization between the two IR segments (Figure 7a). In contrast, the LSC region harbored most of the variability, with multiple discrete Pi peaks exceeding 0.15 (Table 4).

The 15 intergenic spacers with the highest Pi values were identified, with 14 located in the LSC region and one in the IRb region (Figure 7b). The top hypervariable region was located in the *trnM-CAU-rps14* intergenic spacer (Rank 1; position 38,368–38,516 bp; Pi = 0.363), followed by the spacer between *ndhJ* and *ndhK* (Rank 2; Pi = 0.302; position 51,123–51,227 bp) and the spacer between *petL* and *petG* (Rank 3; Pi = 0.301; position 68,300–68,453 bp). Notably, multiple hypervariable regions are flanked by functionally important genes, including *ndhJ*, *ndhK*, *petL*, *petG*, *psbI*, *psbJ*, *rpl33*, *atpI*, *rps14*, *cemA* (Table 4), several of which are involved in photosynthesis or electron transport. Because high nucleotide diversity does not by itself demonstrate barcode performance, these hypervariable intergenic spacers are better described as promising candidate barcode regions requiring further validation for species identification and population genetic studies within *Fraxinus*. In particular, the *ndhJ-ndhK* (Rank 2, Pi = 0.302), *psbI-trnS-GCU* (Rank 6, Pi = 0.211), and *trnM-CAU-rps14* (Rank 1, Pi = 0.363) spacers all show Pi values comparable to or exceeding those reported for the widely used *trnH-psbA* barcode region, making them strong candidates for future barcoding applications in *Fraxinus*.

## 3. Discussion

In this study, we successfully assembled and annotated the complete chloroplast genome of *F. mandshurica* using a hybrid PacBio-Illumina strategy. Compared with approaches relying solely on short-read sequencing, long-read data enabled unambiguous resolution of the complex inverted repeat boundaries and tandem repeat structures, yielding a gap-free circular genome of 155,559 bp. The quadripartite architecture, gene content (132 gene copies; 114 unique genes, including 80 protein-coding genes), and GC content (37.82%) are highly consistent with other *Oleaceae* plastomes, confirming the strong structural conservation of chloroplast genomes within the family. The observation that rRNA genes (55.28% GC) are significantly more GC-rich than protein-coding (37.82%) and tRNA (52.80%) genes is also consistent with the known correlation between high GC content and the structural stability of ribosomal RNA molecules.

SSR analysis revealed that mononucleotide A/T repeats overwhelmingly dominate the six *Fraxinus* cp genomes examined, while di-, tri-, and higher-order repeats are rare or absent. This composition is typical of angiosperm plastomes, where the high AT content of non-coding regions promotes poly-A or poly-T stretch formation through replication slippage. The absence of tri- to hexanucleotide repeats across all six species suggests that higher-order SSRs are under stronger selective constraint in the chloroplast genome, likely due to their greater potential to disrupt gene function when located in coding or regulatory regions. Because our comparison is based on a single plastome per species, the absence of candidate polymorphic SSRs among the six *Fraxinus* species mainly reflects interspecific variation; whether chloroplast SSR markers have discriminatory power at the population or intrasectional level remains to be tested with population-level sampling. Future population genetic studies may benefit from complementing plastid markers with nuclear microsatellites or genome-wide SNP data.

The ML phylogenetic tree reconstructed from 53 complete cp genomes provides robust resolution of relationships within Oleaceae, with all major nodes receiving high bootstrap support [24]. The topology with *J. fluminense* at the most basal position, followed by *S. alata* and then the *Syringa* clade is congruent with recent molecular phylogenetic analyses of the family. Within *Fraxineae*, our data resolve two well-supported lineages in the genus *Fraxinus*. Critically, the placement of *F. mandshurica* as the sister species to the North American endemic *F. nigra* is consistent with previously proposed Miocene Beringian dispersal scenarios, a biogeographic pattern documented in numerous temperate tree genera. Complete plastome sequences provide robust phylogenetic signal; however, they constitute a single, predominantly uniparentally inherited genomic compartment. The phylogenetic framework recovered here should therefore be interpreted as a plastid genealogy rather than a full organismal species tree: chloroplast relationships may not always coincide with the species phylogeny, particularly in groups in which historical introgression or chloroplast capture may have occurred. This caveat is especially relevant to the sister relationship recovered between *F. mandshurica* and *F. nigra*, whose congruence with the nuclear phylogenomic signal warrants confirmation in future genome-wide studies. The stable phylogenetic signal from complete plastome sequences suggests that chloroplast phylogenomics is a powerful tool for resolving deep and shallow relationships within *Fraxinus* [25,26], complementing the limited resolution provided by single-locus markers such as rps16 and trnL-F used in earlier studies.

The Ka/Ks analysis revealed that the vast majority of chloroplast genes in *F. mandshurica* are under strong purifying selection (Ka/Ks << 1), consistent with the essential nature of photosynthetic and translational functions in the chloroplast. However, several genes showed notable deviations from this pattern. Among the single-comparison estimates, *petB* showed the highest Ka/Ks (2.15 in the *F. excelsior* comparison), a value exceeding the neutral threshold of 1.0. Because this value derives from a single species comparison, it is more appropriately interpreted as elevated Ka/Ks suggestive of altered selective constraint than as evidence of positive selection. *petB* encodes a core subunit of the cytochrome b6/f complex, which mediates electron transfer between photosystems II and I. Amino acid changes in this subunit could alter electron transport efficiency, potentially reflecting local adaptation to different light regimes or temperature conditions. The ribosomal protein gene *rpl2* likewise displayed an elevated Ka/Ks (1.17 in the *F. excelsior* comparison); this value is also based on a single species comparison. Among the *ndh* genes, *ndhB* showed consistently elevated Ka/Ks across all three pairwise comparisons, whereas *ndhA* yielded valid estimates in two of them. The NDH complex mediates cyclic electron flow around photosystem I and is critical for maintaining redox balance under environmental stress. The elevated Ka/Ks signal observed in the *ndh* genes is consistent with, although it does not by itself demonstrate, a role in the adaptation of *F. mandshurica* to the cold, high-latitude environments of Northeast China, where efficient photoprotection is essential for survival through prolonged winter dormancy; this hypothesis calls for further functional validation. These findings collectively suggest that while the chloroplast genome is globally conserved, specific functional modules—particularly those involved in electron transport and redox regulation—harbor signatures of adaptive evolution that may contribute to ecological differentiation within *Fraxinus*.

The genome-wide Pi analysis revealed a striking dichotomy in sequence variation across the *F. mandshurica* cp genome. Intergenic spacers in the LSC region were the primary reservoirs of nucleotide diversity, whereas IR regions exhibited essentially zero variation, reflecting the well-known copy-correction mechanism that homogenizes the two IR copies through gene conversion. This pattern of localized hypervariability embedded within a globally conserved genome is consistent with the emerging view that cp genome evolution is characterized by punctuated variation rather than uniform divergence. The identification of the 15 intergenic spacers with the highest Pi values provides a rich resource for marker development in *Fraxinus*. The top three candidates, *trnM-CAU-rps14* (Pi = 0.363), *ndhJ-ndhK* (Pi = 0.302), and *petL-petG* (Pi = 0.301), exhibit Pi values comparable to or exceeding those of the widely used *trnH-psbA* barcode, and their flanking genes are involved in translation initiation (*trnM*), ribosome structure (*rps14*), and electron transport (*ndh*, *pet*), lending support to the hypothesis that regulatory sequences adjacent to functionally critical genes may experience reduced purifying selection. The high Pi observed in the *ndhJ-ndhK* spacer (*N* = 14 species) is particularly promising for species-level barcoding applications, as its broad representation across the genus minimizes the risk of amplification failure in divergent lineages.

## 4. Materials and Methods

### 4.1. Plant Materials and Sequencing

Tender leaves were collected from an adult *F. mandshurica* plus tree located in the Experimental Forest Farm of Northeast Forestry University, Harbin, Heilongjiang Province, China (approximately 45°43′ N, 126°38′ E), in June 2022. A voucher specimen was deposited at the Herbarium of Northeast Forestry University, and the species identity was confirmed by Professor Li Kailong. Genomic DNA for PacBio and Illumina sequencing was prepared from these leaves with a modified CTAB-DNA extraction method followed by QIAGEN Genomic-tip 100/G (Qiagen, Hilden, Germany; 10243) cleaning and filtering. A long fragment PacBio library (insert sizes = 20 kbp) and a short fragment paired-end Illumina library (insert sizes = 500 bp, read length = 250 bp) were prepared and sequenced on the PacBio RS-II platform (PacBio, Menlo Park, CA, USA) and the HiSeq 2500 platform (Illumina, San Diego, CA, USA) at Feisha (Wuhan, China).

### 4.2. Data Filtration

To remove adaptor and low-quality reads, raw Illumina data were filtered using fastp tool v0.13.1 (trim reads from 5′ = 10, trim reads from 3′ = 20, qualified quality phred = Q20, unqualified percent limit = 20%) [27]. Clean reads were also checked using FastQC (v.0.11.8) [28] tool to make sure all these satisfied the quality-control requirements.

### 4.3. Genome Assembly, Annotation, and Visualization

To complete the assembly, the chloroplast genome sequence of *Fraxinus excelsior* (GenBank: MG214254.1) was used as a guide. A total of 66.78 Gb of PacBio subreads (N50 = 11,847 bp) was generated for assembly. All sequence data generated that were derived from PacBio RS-II platform were assembled with Organelle_PBA pipeline [29]. The Illumina reads were mapped onto the initial cp genome of *F. mandshurica* using BWA [30] software (v. 0.7.17) with mem algorithm. Pilon (v.1.23) was then used to polish the initial assembly [31]. This procedure was repeated until no correction occurred.

We used the online Chloroplast Genome Annotation, Visualization, Analysis and GenBank Submission Tool 2 (CPGAVAS2) to generate the draft annotations [32,33]. All tRNA genes were predicted by tRNAscan-SE 2.0 [34]. We finally corrected the draft annotations by using the Apollo Genome Annotation and Curation Tool (v1.11.8) [35] manually. The corrected chloroplast annotation was submitted to CPGAVAS2 for re-analysis.

The mean sequencing depth of the chloroplast genome was approximately 7043×, with 1.58% of the total Illumina reads mapping to the final assembly. The IR/LSC and IR/SSC junctions and the circular conformation of the plastome were directly confirmed by PacBio long reads spanning these boundaries.

### 4.4. Codon Usage Statistics

We analyzed the codon usage for all protein-coding genes. Although RNA editing may occur at some sites, we ignored them here. MEGA X [36] software (version 10.1.6) was used to calculate the codon usage and the relative synonymous codon usage (RSCU) value. Because the genetic code of the chloroplast genome differs from the standard code, Plant Plastid was selected as the genetic code in MEGA X.

Genes duplicated in the IR regions were retained in the analysis because they are actively transcribed in the plastome and contribute to the overall codon pool; this approach is consistent with previous codon-usage surveys of Oleaceae plastomes. Genes with non-standard initiation codons (*ndhD*, *rps19*, and *ycf15*) were analyzed using the plastid genetic code, in which these alternative start codons are translated as methionine. RNA editing was not incorporated; consequently, the codon-usage and RSCU values are based on unedited RNA sequences, which may slightly affect the values at edited sites.

### 4.5. Genome Comparison

The complete cp genome sequences of *F. mandshurica* and five other species, *F. americana* (NC_042449.1), *F. chiisanensis* (MF980720.1), *F. chinensis* (MK299391.1), *F. quadrangulata* (NC_042451.1), *F. xanthoxyloides* (NC_042452.1) from different sections of the *Fraxinus* genus, were aligned using the program mVISTA [37]. The SLAGAN algorithm with translated anchoring was used to form VISTA plots. The RankVISTA probability threshold was set at 0.5 to obtain accurate identity and similarity.

### 4.6. SSR Analysis

SSR identification was performed using the Perl script MISA [38] (MIcroSAtellite identification tool) and categorized using standard parameters (the minimum number of repeats of 10, 5, 4, 3, 3 and 3 for mono-, di-, tri-, tetra-, penta- and hexanucleotides, respectively). Meanwhile, CandiSSR [39], an efficient pipeline used for identifying candidate polymorphic SSRs based on multiple assembled sequences, was used to identify PolySSRs and to automatically design primer pairs for each identified PolySSR in *F. mandshurica* and five other species from the genus *Fraxinus* above.

### 4.7. Analysis of IR Expansion and Contraction

To examine expansion or contraction of the IR regions for *Oleaceae* taxa, four species *Syringa oblata* (MT025818.1), *Schrebera alata* (NC_042467.1), *Fraxinus mandshurica* and *Olea europaea* (GU931818.1) were selected to represent the Subtribes *Ligustrinae*, *Schreberinae*, *Fraxininae* and *Oleinae*, respectively, depending on the APG (Angiosperm Phylogeny Group) IV system of flowering plant classification. These four taxa were selected as a representative comparison to illustrate the range of IR/SC boundary configurations across the four Oleaceae subtribes (*Ligustrinae*, *Schreberinae*, *Fraxininae*, and *Oleinae*), rather than to support broad evolutionary conclusions.

### 4.8. Phylogenetic Analysis

The phylogenetic analysis was based on the complete chloroplast genome sequence of *F. mandshurica* and 52 other dicotyledonous species downloaded from the NCBI database (https://www.ncbi.nlm.nih.gov/; accessed on 15 August 2025). These downloaded sequences comprise representative species from the family Oleaceae, primarily belonging to the genera *Fraxinus* and *Syringa*. Furthermore, five distantly related species, namely *Arabidopsis thaliana*, *Panax ginseng*, *Eleutherococcus senticosus*, *Solanum lycopersicum*, and *Nicotiana tabacum*, were included and designated as outgroups. The whole chloroplast genome sequences were aligned using MAFFT [40]. To remove poorly aligned regions and spurious sequences, the alignments were subsequently trimmed using trimAl [41]. The program IQ-TREE [42] was employed to automatically find the optimal nucleotide substitution model via ModelFinder [43] and to reconstruct a maximum likelihood phylogenetic tree. Ultrafast bootstrap resampling with 1000 replicates was used to evaluate the branch supports.

The best-fit nucleotide substitution model selected by ModelFinder under the Bayesian information criterion was TVM + F + I + R4, and the final trimmed alignment comprised 155,234 bp from 53 chloroplast genomes.

### 4.9. Selection Pressure Analysis

To evaluate the evolutionary selection pressure acting on protein-coding genes of the *F. mandshurica* chloroplast genome, we calculated the ratio of nonsynonymous to synonymous substitutions (Ka/Ks) using the Nei–Gojobori method as implemented in custom Python scripts (Biopython v1.88 [44]). For each of the 80 unique protein-coding genes identified in the *F. mandshurica* cp genome, orthologous coding sequences (CDS) were extracted from three closely related *Fraxinus* species: *F. nigra* (NC_042450.1), *F. excelsior* (MG214254.1), and *F. americana* (NC_042449.1). As chloroplast genomes are highly collinear within the genus *Fraxinus*, orthologous CDS were localized using the conserved gene coordinates of the *F. mandshurica* reference genome. Genomic sequences were aligned using the Needleman–Wunsch algorithm at the protein level, and codon alignments were reconstructed accordingly. Synonymous (Ks) and nonsynonymous (Ka) substitution rates were calculated for each orthologous gene pair, with the Jukes–Cantor correction applied to account for multiple substitutions. A gene was excluded from a given comparison (recorded as N/A) when the estimated Ks was zero (no synonymous substitutions, rendering Ka/Ks undefined), when the gene was absent or pseudogenized in the compared species, or when the aligned region contained too few informative sites to reliably estimate substitution rates. Genes were classified as evolving under purifying selection (Ka/Ks < 1), neutral evolution (Ka/Ks ≈ 1), or positive/diversifying selection (Ka/Ks > 1). Pairwise Ka/Ks estimates were interpreted descriptively. Genes with an average Ka/Ks of 0.8 or higher across valid comparisons were highlighted, when supported by at least two species comparisons; estimates based on a single comparison were reported separately with caution. No pairwise Ka/Ks value was considered, by itself, evidence of positive selection. Table 3 reports the genes with interpretable pairwise estimates, including several below the 0.8 threshold; the complete per-comparison estimates are summarized in Figure 6.

### 4.10. Nucleotide Diversity Analysis

To identify hypervariable regions with potential utility as molecular markers or DNA barcodes within the genus *Fraxinus*, we computed nucleotide diversity (Pi, π) across 57 intergenic spacer regions of the cp genome. Complete cp genome sequences of 15 *Fraxinus* species (*F. americana*, *F. angustifolia*, *F. chiisanensis*, *F. chinensis*, *F. excelsior*, *F. insularis*, *F. lanuginosa*, *F. latifolia*, *F. mandshurica*, *F. nigra*, *F. pennsylvanica*, *F. quadrangulata*, *F. sieboldiana*, *F. velutina*, and *F. xanthoxyloides*) were retrieved from NCBI. For each intergenic spacer of the *F. mandshurica* cp genome (as annotated by CPGAVAS2), homologous sequences were extracted from each species using coordinate-based mapping followed by local alignment refinement within ±500 bp of the expected position. Each set of orthologous intergenic sequences was globally aligned using the Needleman–Wunsch algorithm (Biopython v1.88), and nucleotide diversity was calculated per region as the average number of pairwise nucleotide differences per site. The 15 intergenic spacers with the highest Pi values were considered hypervariable.

## 5. Conclusions

In this study, we assembled and characterized a complete, fully annotated chloroplast genome of *F. mandshurica* through an integrated PacBio-Illumina sequencing approach. The 155,559 bp genome exhibits the conserved quadripartite structure and gene repertoire characteristic of *Oleaceae* plastomes. Our multi-faceted comparative and evolutionary analyses yielded several key findings: (1) the phylogenetic reconstruction based on 53 complete cp genomes robustly resolved intergeneric relationships within *Oleaceae* and identified *F. mandshurica* as the sister species to the North American *F. nigra*, consistent with previously proposed Miocene Beringian dispersal scenarios for temperate tree taxa; (2) selection pressure analysis revealed that most plastid protein-coding genes are under strong selective constraint, whereas a small subset, including *petB*, *rpl2*, and several *ndh* genes, exhibited comparatively elevated pairwise Ka/Ks estimates; because some estimates were based on very few substitutions or only one valid species comparison, these patterns should be regarded as exploratory evidence of altered selective constraint rather than as evidence of positive selection; (3) genome-wide nucleotide diversity analysis identified 15 hypervariable intergenic spacers, with *trnM-CAU-rps14*, *ndhJ-ndhK*, and *petL-petG* emerging as the most promising candidate barcode regions, which nevertheless require further validation before their practical utility for species identification can be confirmed; and (4) SSR profiling confirmed the dominance of mononucleotide A/T repeats but revealed limited interspecific polymorphism, underscoring the need for complementary nuclear markers in population-level studies. Collectively, this work substantially expands the genomic toolkit available for *Fraxinus* and provides a foundational resource for future studies on the molecular evolution, phylogenomics, conservation genetics, and sustainable utilization of this ecologically and economically important hardwood genus.

## Figures and Tables

**Table 1:** Group of genes within *F. mandshurica* cp genome.

Category for Genes	Group of Genes	Gene Names
Photosynthesis	Rubisco large subunit	*rbcL*
	Photosystem I	*psaA*, *psaB*, *psaC*, *psaI*, *psaJ*
	Assembly/stability of Photosystem I	*ycf3*, *ycf4*
	Photosystem II	*psbA*, *psbB*, *psbC*, *psbD*, *psbE*, *psbF*, *psbH*, *psbI*, *psbJ*, *psbK*, *psbL*, *psbM*, *psbN*, *psbT*, *psbZ/ycf9*
	ATP synthase	*atpA*, *atpB*, *atpE*, *atpF*, *atpH*, *atpI*
	Cytochrome b/f complex	*petA*, *petB*, *petD*, *petG*, *petL*, *petN/ycf6*
	Cytochrome c synthesis	*ycf5/ccsA*
	NADPH dehydrogenase	*ndhA*, *ndhB*, *ndhC*, *ndhD*, *ndhE*, *ndhF*, *ndhG*, *ndhH*, *ndhI*, *ndhJ*, *psbG/ndhK*
Transcription and translation	RNA polymerase	*rpoA*, *rpoB*, *rpoC1*, *rpoC2*
	Ribosomal proteins (SSU)	*rps2*, *rps3*, *rps4*, *rps7*, *rps8*, *rps11*, *rps12*, *rps14*, *rps15*, *rps16*, *rps18*, *rps19*
	Ribosomal proteins (LSU)	*rpl2*, *rpl14*, *rpl16*, *rpl20*, *rpl22*, *rpl23*, *rpl32*, *rpl33*, *rpl36*
	Translation initiation factor	*infA*
RNA genes	Ribosomal RNAs	*rrn4.5S*, *rrn5S*, *rrn16S*, *rrn23S*
	Transfer RNAs	*trnA-UGC*, *trnC-GCA*, *trnD-GUC*, *trnE-UUC*, *trnF-GAA*, *trnfM-CAU*, *trnG-GCC*, *trnG-UCC*, *trnH-GUG*, *trnI-AUG*, *trnI-GAU*, *trnK-UUU*, *trnL-CAA*, *trnL-UAA*, *trnL-UAG*, *trnM-CAU*, *trnN-GUU*, *trnP-UGG*, *trnQ-UUG*, *trnR-ACG*, *trnR-UCU*, *trnS-GCU*, *trnS-GGA*, *trnS-UGA*, *trnT-GGU*, *trnT-UGU*, *trnV-GAC*, *trnV-UAC*, *trnW-CCA*, *trnY-GUA*,
others	Cell survival	*ycf1*, *ycf2*
	RNA processing	*matK*
	Envelope membrane protein	*cemA/ycf10*
	Subunit of Acetyl-CoA-carboxylase	*accD*
	Proteolysis	*clpP*
	Unknown function	*ycf15*

**Table 2:** RSCU value of the *F. mandshurica* cp genome.

Codon	AA	Count	RSCU	RSCU ^Fs^	RSCU ^Nt^	Codon	AA	Count	RSCU	RSCU ^Fs^	RSCU ^Nt^
**UUU**	Phe	946	1.27	1.32	1.28	UAU	Tyr	783	1.61	1.61	1.61
**UUC**	Phe	547	0.73	0.68	0.72	UAC	Tyr	188	0.39	0.39	0.39
**UUA**	Leu	847	1.82	1.93	1.86	UAA	*	43	1.48	1.62	1.59
**UUG ^#^**	Leu	571	1.23	1.21	1.21	UAG	*	22	0.76	0.58	0.79
**CUU**	Leu	609	1.31	1.30	1.31	CAU	His	496	1.57	1.58	1.55
**CUC**	Leu	181	0.39	0.35	0.44	CAC	His	136	0.43	0.42	0.45
**CUA**	Leu	386	0.83	0.81	0.78	CAA	Gln	721	1.52	1.52	1.50
**CUG ^#^**	Leu	191	0.41	0.40	0.40	CAG	Gln	226	0.48	0.48	0.50
**AUU**	Ile	1097	1.47	1.47	1.46	AAU	Asn	990	1.55	1.56	1.52
**AUC**	Ile	470	0.63	0.62	0.61	AAC	Asn	289	0.45	0.44	0.48
**AUA**	Ile	679	0.91	0.91	0.92	AAA	Lys	1041	1.48	1.54	1.47
**AUG ^#^**	Met	630	1.00	1.00	1.00	AAG	Lys	362	0.52	0.46	0.53
**GUU**	Val	525	1.47	1.48	1.46	GAU	Asp	878	1.62	1.59	1.60
**GUC**	Val	176	0.49	0.44	0.50	GAC	Asp	206	0.38	0.41	0.40
**GUA**	Val	536	1.50	1.56	1.50	GAA	Glu	1042	1.51	1.54	1.49
**GUG**	Val	192	0.54	0.53	0.54	GAG	Glu	341	0.49	0.46	0.51
**UCU**	Ser	599	1.73	1.76	1.75	UGU	Cys	230	1.58	1.53	1.48
**UCC**	Ser	318	0.92	0.92	0.95	UGC	Cys	61	0.42	0.47	0.52
**UCA**	Ser	425	1.23	1.15	1.18	UGA	*	22	0.76	0.81	0.62
**UCG**	Ser	194	0.56	0.57	0.58	UGG	Trp	469	1.00	1.00	1.00
**CCU**	Pro	439	1.57	1.55	1.52	CGU	Arg	341	1.26	1.30	1.28
**CCC**	Pro	209	0.75	0.75	0.76	CGC	Arg	102	0.38	0.42	0.36
**CCA**	Pro	327	1.17	1.19	1.18	CGA	Arg	364	1.35	1.34	1.46
**CCG**	Pro	147	0.52	0.50	0.54	CGG	Arg	133	0.49	0.46	0.46
**ACU**	Thr	544	1.62	1.63	1.55	AGU	Ser	422	1.22	1.26	1.19
**ACC**	Thr	250	0.74	0.76	0.78	AGC	Ser	115	0.33	0.34	0.35
**ACA**	Thr	405	1.20	1.23	1.22	AGA	Arg	505	1.87	1.85	1.78
**ACG**	Thr	148	0.44	0.38	0.46	AGG	Arg	178	0.66	0.63	0.66
**GCU**	Ala	621	1.81	1.84	1.78	GGU	Gly	584	1.29	1.33	1.25
**GCC**	Ala	212	0.62	0.62	0.70	GGC	Gly	171	0.38	0.39	0.46
**GCA**	Ala	402	1.17	1.14	1.13	GGA	Gly	740	1.63	1.60	1.59
**GCG**	Ala	140	0.41	0.40	0.39	GGG	Gly	321	0.71	0.68	0.70

^#^ is a start codon. The most common start codon is AUG. But UUG and CUG are alternative start codons in the plastid; they are still translated as Met when they are at the start of a protein (even if the codon encodes a different amino acid otherwise). * is a stop codon. ^Fs^ is the RSCU value of the *Forsythia suspensa* (a species of *Oleaceae*) cp genome and ^Nt^ is the value of the *Nicotiana tabacum* (Z00044.2) cp genome.

**Table 3:** Selection pressure (Ka/Ks) analysis of *F. mandshurica* chloroplast genes.

Gene	Annotation	Avg Ka	Avg Ks	Avg Ka/Ks	vs. *F. nigra*	vs. *F. excelsior*	vs. *F. americana*	N spp.
*petB*	Cytochrome b6/f	0.0072	0.0033	2.152	N/A	2.152	N/A	1
*rpl2*	Ribosomal protein (large)	0.0395	0.0338	1.1674	N/A	1.1674	N/A	1
*rps16*	Ribosomal protein (small)	0.2973	0.2871	0.9762	N/A	0.9153	1.0371	2
*clpP*	Protease	0.3745	0.3918	0.9751	N/A	1.0445	0.9058	2
*ndhA*	NADH dehydrogenase	0.4758	0.549	0.8515	N/A	0.8175	0.8855	2
*ycf3*	Hypothetical (ycf3)	0.4605	0.5567	0.8271	N/A	0.8271	N/A	1
*ndhB*	NADH dehydrogenase	0.4717	0.6423	0.7761	0.8123	0.8542	0.6617	3
*rpoC1*	RNA polymerase	0.3321	0.572	0.7198	0.5795	0.86	N/A	2
*ycf1*	Hypothetical (ycf1)	0.5208	0.7369	0.7011	0.7822	0.62	N/A	2
*atpF*	ATP synthase	0.5326	1.0596	0.5026	0.5026	N/A	N/A	1
*petL*	Cytochrome b6/f	0.3363	0.5409	0.3109	0.6217	0.0	N/A	2
*ndhD*	NADH dehydrogenase	0.0009	0.0029	0.298	N/A	0.298	N/A	1
*matK*	Maturase	0.0017	0.0061	0.2766	0.2766	N/A	N/A	1
*rps2*	Ribosomal protein (small)	0.0018	0.0066	0.2754	N/A	0.2754	N/A	1
*rpoC2*	RNA polymerase	0.0013	0.0054	0.2257	0.2887	0.0721	0.3162	3

N/A, not applicable: Ka/Ks was not computed for a comparison where Ks = 0 (no synonymous substitutions), where no reliable orthologous alignment was available, or where the aligned region contained too few informative sites to reliably estimate substitution rates.

**Table 4:** Top 15 hypervariable intergenic regions in the *F. mandshurica* chloroplast genome.

Rank	Start (bp)	End (bp)	Length (bp)	Pi	N Species	Upstream Gene	Downstream Gene	Region
1	38,368	38,516	149	0.362906	9	*trnM-CAU*	*rps14*	LSC
2	51,123	51,227	105	0.302039	14	*ndhJ*	*ndhK*	LSC
3	68,300	68,453	154	0.301139	10	*petL*	*petG*	LSC
4	54,100	54,279	180	0.234461	12	*trnV-UAC*	*trnM-CAU*	LSC
5	11,130	11,241	112	0.211706	10	*trnR-UCU*	*atpA*	LSC
6	9241	9364	124	0.210827	13	*psbI*	*trnS-GCU*	LSC
7	68,568	68,692	125	0.210099	11	*petG*	*trnW-CCA*	LSC
8	70,207	70,385	179	0.206526	11	*rpl33*	*rps18*	LSC
9	32,150	32,264	115	0.199726	12	*trnD-GUC*	*trnY-GUA*	LSC
10	66,353	66,485	133	0.192553	7	*psbJ*	*psbL*	LSC
11	153,580	153,744	165	0.187566	10	*trnI-AUG*	*rpl23*	IRb
12	16,478	16,700	223	0.165449	7	*atpI*	*rps2*	LSC
13	10,892	11,057	166	0.164893	9	*trnG-UCC*	*trnR-UCU*	LSC
14	64,113	64,339	227	0.126285	4	*cemA*	*petA*	LSC
15	54,353	54,557	205	0.125532	9	*trnM-CAU*	*atpE*	LSC

## Data Availability

The data presented in this study are openly available in the National Center for Biotechnology Information (NCBI) GenBank database at https://www.ncbi.nlm.nih.gov; accessed on 18 August 2026, reference numbers PZ871292.
